# Change of the Color of the Hair in a Night; Report of a Case

**Published:** 1864-10

**Authors:** J. F. Miner


ART. III.—*Change of the Color of the Hair in a night; report of a case.* BY J. F. MINER, M. D.

The following case of change in the color of hair, supposed to arise from violent mental emotion is regarded as of sufficient interest to be worthy of record:

John S. Constantine, aged 33, from Colden, Erie county, N. Y., now member of the 14th Reg't N. Y. Heavy Artillery, was transferred to the Buffalo General Hospital July 14, 1864, with gun-shot wound of foot, and gives the following account of the change of color observed in the hair upon the right side of the head: "During the winter of 1846, when 16 years of age, I was abruptly informed of the death of my mother. As I had just buried a sister, to hear of my mother's death made considerable impression upon my mind, though nothing that I regarded as unusual at the time. I was away from home and could not return until next day. When I did arrive I found my mother alive and better, and she soon inquired of me what was the matter of my hair; upon examination I found the hair upon the right half of my scalp white. Have always enjoyed good health, and suffered in no other way except change in the color of hair."

Mr. Constantine is an intelligent, healthy looking young man, and the account he gives of himself is undoubtedly correct. A large portion of the hair upon one side of the head is white, the remainder being of a dark brown color; one eye-brow has also a tuft of white hair. He has been in the army near a year; since wounded has been in several hospitals, and been observed by a great many surgeons and medical men. He says, "All say they have heard of such things, but never saw a case before." There are also patches of skin which are unnaturally white; these he says never tan or grow brown by exposure.

I remember, in my school-days, having read in my lessons in Wayland's Moral Science, similar accounts of change of hair in a single night, from violent mental emotion, but had nearly come to regard Wayland's Moral Science as probably mistaken, when attention was attracted to the condition of the hair upon the soldier's head.

Here are unquestionably the facts; who will give the explanation? Possibly Dr. John O'Reilly of New York, who has written a most ingenious and philosophical work upon the functions of the nervous system, would inform us that this effect was produced through the organic nervous system. He says, "When grief or anxiety harrasses the mind, the organic nervous system sympathises and fully participates in the troubles of the mind. The painful sensation and oppression experienced about the heart, in popular language, is the result of the communication established between the superior central ganglion, and the semilunar ganglions, through the branches of the par vagi, which inosculate with the branches of the solar plexus and the branches of the cardiac plexus." Possibly he might give some such explanation of the manner of communication between the hair and brain, and it is perhaps as good as any which can be given. It is however a very remarkable fact that the coloring matter which was in the hair should so soon become faded or bleached. If from any nervous influence it should cease to be furnished with coloring material would not be quite so remarkable; that it should disappear in a night is inexplicable.

Melanine, the coloring matter which is found in the choroid coat of the eye, the iris, and the skin, is the same as that found in the hair, and is said to be insoluble in water and the dilute acids, but dissolves slowly in caustic potassa; by what influence it is ever so completely and suddenly removed or changed I do not propose at present to determine.

